# Supplementary material for: Use of Electrical Impedance Tomography (EIT) to Estimate Tidal Volume in Anaesthetized Horses Undergoing Elective Surgery
Source: Animals (Basel). 2021 May 10;11(5):1350. doi: 10.3390/ani11051350 (PMC8151473; doi:10.3390/ani11051350)
Supplement: Supplementary file 1 [file animals-11-01350-s001.zip › Supplementary Table S1.docx]

**Table S1** Demographic data for each of the 17 anaesthetised horses, including breed, sex, age, weight, chest circumference and body condition score.

| Horse | Breed | Sex | Age (Months) | Weight (Kg) | Chest circumference  (cm) | Body Condition Score (BCS) |
| --- | --- | --- | --- | --- | --- | --- |
| 1 | Thoroughbred | M | 23 | 490 | 184 | 6/9 |
| 2 | Warmblood | M | 23 | 480 | 180 | 5/9 |
| 3 | Warmblood | F | 36 | 471 | 180 | 4/9 |
| 4 | Stock Horse | M | 157 | 588 | 212 | 5/9 |
| 5 | Thoroughbred | MG | 36 | 498 | 181 | 4/9 |
| 6 | Thoroughbred | M | 24 | 448 | 178 | 5/9 |
| 7 | Standardbred | M | 36 | 396 | 165 | 5/9 |
| 8 | Standardbred | M | 48 | 479 | 178 | 4/9 |
| 9 | Standardbred | M | 24 | 434 | 173 | 6/9 |
| 10 | Thoroughbred | MG | 36 | 552 | 195 | 5/9 |
| 11 | Standardbred | M | 42 | 429 | 173 | 5/9 |
| 12 | Thoroughbred | F | 68 | 431 | 181 | 5/9 |
| 13 | Thoroughbred | M | 47 | 482 | 180 | 5/9 |
| 14 | Thoroughbred | M | 227 | 540 | 190 | 6/9 |
| 15 | Quarter Horse | F | 84 | 542 | 194 | 5/9 |
| 16 | Thoroughbred | M | 60 | 527 | 182 | 5/9 |
| 17 | Standardbred | M | 62 | 460 | 172 | 5/9 |
| M, male entire; MG, male gelded; F, female; RF, right forelimb; LF, left forelimb; RH, right hindlimb; LH, left hindlimb; | | | | | | |
