# Supplementary material for: Use of Electrical Impedance Tomography (EIT) to Estimate Tidal Volume in Anaesthetized Horses Undergoing Elective Surgery
Source: Animals (Basel). 2021 May 10;11(5):1350. doi: 10.3390/ani11051350 (PMC8151473; doi:10.3390/ani11051350)
Supplement: Supplementary file 1 [file animals-11-01350-s001.zip › Supplementary Table S3.docx]

**Table S3.** Intercept and slope for simple linear regression between impedance measured using EIT (∆Z_breath_) and tidal volume measured using spirometry (VT_SPIRO_) at measurement point M_10_, M_12_ and M_15_.

| Horse | Intercept  (SE) | Slope  (SE) | R2 |
| --- | --- | --- | --- |
| 1 | -0.07  (0.53) | 0.71  (0.08) | 0.987 |
| 2 | 0.13  (0.10) | 0.46  (0.02) | 0.998 |
| 3 | 1.12  (0.08) | 0.37  (0.01) | 0.999 |
| 4 | 0.20  (0.17) | 0.56  (0.02) | 0.998 |
| 5 | 0.32  (0.13) | 0.33  (0.02) | 0.996 |
| 6 | 1.26  (0.31) | 0.26  (0.05) | 0.963 |
| 7 | 1.66  (0.01) | 0.35  (0.003) | 0.999 |
| 8 | 0.27  (0.41) | 0.55  (0.07) | 0.986 |
| 9 | 0.66  (0.22) | 0.56  (0.04) | 0.995 |
| 10 | 0.62  (0.10) | 0.35  (0.01) | 0.998 |
| 11 | 0.13  (0.05) | 0.68  (0.01) | 0.999 |
| 12 | -0.08  (0.22) | 0.63  (0.05) | 0.994 |
| 13 | 0.39  (0.20) | 0.46  (0.04) | 0.994 |
| 14 | -0.82  (0.64) | 0.70  (0.09) | 0.983 |
| 15 | 0.54  (0.11) | 0.51  (0.02) | 0.999 |
| 16 | 0.05  (0.02) | 0.41  (0.003) | 1.00 |
| 17 | 0.03  (0.04) | 0.61  (0.01) | 0.999 |
