# Supplementary material for: Use of Electrical Impedance Tomography (EIT) to Estimate Tidal Volume in Anaesthetized Horses Undergoing Elective Surgery
Source: Animals (Basel). 2021 May 10;11(5):1350. doi: 10.3390/ani11051350 (PMC8151473; doi:10.3390/ani11051350)
Supplement: Supplementary file 1 [file animals-11-01350-s001.zip › Supplementary Table S4.docx]

**Table S4.** Estimated tidal volume (VT_EIT_) from ∆Z_breath_ at measurement M_A_, M_B_ and M_C_ for each horse using the intercept and slope of line of best fit extrapolated from the ∆Z_breath_ and VT_SPIRO_ relationship determined at measurement M_10_, M_12_ and M_15_. Tidal volume (VT_SPIRO_) measured using spirometry is presented for comparison.

| HORSE | Measurement Point | Measured VT_SPIRO_ (L) | Estimated VT_EIT_ (L) |
| --- | --- | --- | --- |
| 1 | M_A_ | 7.24 | 7.20 |
|  | M_C_ | 7.10 | 7.05 |
| 2 | M_A_ | 7.18 | 7.22 |
|  | M_B_ | 7.12 | 7.31 |
| 3 | M_A_ | 7.43 | 7.61 |
|  | M_B_ | 7.53 | 7.78 |
| 4 | M_A_ | 7.35 | 6.90 |
|  | M_B_ | 7.52 | 6.97 |
| 5 | M_A_ | 7.39 | 6.80 |
|  | M_B_ | 7.39 | 6.51 |
|  | M_C_ | 7.44 | 6.15 |
| 6 | M_A_ | 7.68 | 7.68 |
| 7 | M_A_ | 6.39 | 5.65 |
| 8 | M_A_ | 7.06 | 6.97 |
|  | M_B_ | 7.08 | 6.93 |
| 9 | M_A_ | 6.26 | 5.79 |
| 10 | M_A_ | 8.14 | 7.96 |
| 11 | M_B_ | 6.52 | 6.10 |
|  | M_C_ | 6.61 | 6.01 |
| 12 | M_A_ | 6.49 | 6.23 |
|  | M_B_ | 6.42 | 6.20 |
|  | M_C_ | 6.54 | 6.20 |
| 13 | M_A_ | 6.34 | 6.40 |
|  | M_B_ | 6.34 | 6.29 |
| 14 | M_A_ | 7.46 | 7.17 |
|  | M_B_ | 7.32 | 6.94 |
| 15 | M_A_ | 7.25 | 7.16 |
|  | M_B_ | 7.46 | 7.35 |
|  | M_C_ | 7.46 | 7.23 |
| 16 | M_A_ | 7.44 | 7.33 |
| 17 | M_A_ | 6.93 | 6.51 |
|  | M_B_ | 6.85 | 6.43 |
