# Supplementary material for: Use of Electrical Impedance Tomography (EIT) to Estimate Tidal Volume in Anaesthetized Horses Undergoing Elective Surgery
Source: Animals (Basel). 2021 May 10;11(5):1350. doi: 10.3390/ani11051350 (PMC8151473; doi:10.3390/ani11051350)
Supplement: Supplementary file 1 [file animals-11-01350-s001.zip › Supplementary TableS2.docx]

**Table S2** Surgical procedures performed, and anaesthetic protocol tailored for each of the 17 horses. The endotracheal tube (ETT) size is also indicated.

| Horse | Procedure | Premedication | Induction | Perioperative Drugs | ETT |
| --- | --- | --- | --- | --- | --- |
| 1 | Closed Castration + Hernia repair | Xyla | Keta, Midaz | Metha, Medet CRI, Lido, Dbt | 26 |
| 2 | Closed  Castration | Ace; Xyla | Keta, Diaz | Morph, Medet CRI, Dbt | 26 |
| 3 | Sequestrum  removal | Ace; Xyla | Keta, Diaz | Morph | 24 |
| 4 | Closed  Castration | Xylazine | Keta, Diaz | Morph | 26 |
| 5 | RF Fetlock Arthroscopy | Xylazine | Keta, Diaz | Morph, Medet CRI, Dbt | 26 |
| 6 | Closed  Castration | Ace; Xyla | Keta, Diaz | Morph, Medet CRI, Dbt | 26 |
| 7 | Closed  Castration | Ace; Xyla | Keta, Diaz | Morph, Medet CRI, Dbt, Mepi IT | 26 |
| 8 | Closed  Castration | Ace; Xyla | Keta, Diaz | Morph, Dbt, Mepi IT | 26 |
| 9 | Closed  Castration | Ace; Xyla | Keta, Diaz | Morph, Medet CRI, Dbt, Mepi IT | 24 |
| 10 | RF Fetlock Arthroscopy | Ace, Romif | Keta, Diaz | Morph, Medet CRI | 26 |
| 11 | LH stifle cyst  removal | Ace, Xyla | Keta, Diaz | Morph, Romif CRI | 24 |
| 12 | R Carpal  Arthroscopy | Xyla | Keta, Diaz | Morph, Medet CRI, Dbt | 26 |
| 13 | Bilateral carpal arthroscopy | Xyla | Keta, Diaz | Medet CRI, Dbt | 26 |
| 14 | R Carpal Arthroscopy + Wound debridement | Xyla | Keta, Diaz, Thio | Medet CRI, Dbt | 30 |
| 15 | LH Stifle  Arthroscopy | Ace; Xyla; | Keta, Diaz | Medet CRI, Dbt | 26 |
| 16 | RH Fetlock Arthroscopy | Ace; Xyla; | Keta, Diaz | Morph, Romif CRI, Dbt | 30 |
| 17 | Closed  Castration | Ace; Xyla; | Keta, Diaz | Morph, Medet CRI | 26 |
| Xyla, xylazine; Romif, romifidine; Ace, acepromazine; Keta, ketamine; Midaz, midazolam; Diaz, diazepam; Metha, methadone; Morph, morphine; Medet, medetomidine; Lido, Lidocaine, Dbt, dobutamine; Mepi IT, intra-testicular mepivacaine; CRI, constant-rate infusion | | | | | |
